# Evolution Process of Fault Silica Aerogel under High Temperatures: A Molecular Dynamics Approach

**DOI:** 10.3390/gels10080539

**Published:** 2024-08-20

**Authors:** Wenping Yue, Tao Luo, Kaide Liu

**Affiliations:** Shaanxi Key Laboratory of Safety and Durability of Concrete Structures, The Youth Innovation Team of Shaanxi Universities, College of Civil Engineering, Xijing University, Xi’an 710123, China; 20160146@xijing.edu.cn (T.L.); liukaide@xijing.edu.cn (K.L.)

**Keywords:** silica aerogel, faults, fireproof, high temperature, molecular dynamics

## Abstract

Building fire will seriously threaten human safety. Silica aerogel with low thermal conductivity and thermal stability as fire-retardant material has been widely used in building fireproof structures. However, the natural fragility of silica aerogel will limit its application. In this work, the effects of faults on the thermal stability of silica aerogel are studied by molecular dynamics simulation with large simulation time (20 ns). Additionally, the atomic model of silica aerogel with random faults is built by a straining structure (tensile strains are 10%, 20%, 30%, and 40%). It is found that when the tensile strain is less than 20%, the silica backbone can remain stable. The effects of faults on the thermal stability can be neglected. The silica backbone thermally vibrates during the heating process. However, when the tensile strain is over 30%, it is observed that the faults will enhance the silica backbone merging. Silica aerogel can be stable under 800 K. It is believed that the results of this study will pave the way for the development of fireproof materials.

## 1. Introduction

Silica nanoparticle-based materials possess high thermal insulation, fire resistance, and chemical stability, which have been widely used in the application of building engineering [1,2,3,4]. Here, silica aerogel is synthesized using the sol–gel method [5,6,7,8,9]. It leads to silica aerogel with the lowest thermal conductivity (around 0.01 W/mK), a high specific area, and high thermal stability [10,11,12,13,14]. These significant structural and physical properties are attributed to its three-dimensional backbone. The solid silica backbone is formed by the aggregated silica nanoparticles. It will generate limited connectivity and limit the solid thermal conduction. Additionally, this natural solid backbone will provide rich pore structures with a few nanometers of gas molecules transferring. It makes the gas molecules transferring at a non-continuous state [10], limiting the gases’ thermal conduction. This unique solid backbone of silica aerogel makes silica aerogel the candidate with best potential for building engineering and energy saving [15,16,17].

With the development of nanomaterials and nanotechnologies, phase change materials (PCMs) are combined with or incorporated into the nanomaterial as aerogel composites in the application of building engineering [18,19,20,21]. Silica aerogel with a high specific area and flame-retardant properties acts as an ideal carrier of PCMs [20,22]. For example, Liu et al. [23] proposed a silica aerogel composite with low absorption capability and high thermal insulation for building insulation. Lee et al. [24] fabricated flexible and thermally insulated composites by using a flexible poly(dimethylsiloxane) (PDMS) matrix and by preventing matrix impregnation of the silica aerogel pores as thermal insulation materials with flexibility and flame-retardant properties [25,26,27,27]. Zhou et al. [28] prepared a novel PCM using the PCM as PCM and silica aerogels as carriers in energy-efficient buildings. Despite the aerogel’s inherent properties of ultralight weight, ultrahigh porosity, ultrahigh specific area, and lowest thermal conductivity, the natural fragility of silica aerogel limits its applications in building engineering. In other words, the structure of silica aerogel will no doubt be damaged. Therefore, the thermal stability of silica aerogel with faults is the basement as an efficient-building material. When a fire occurs in a building, some of the phase change organic materials or thermal insulation materials will release toxic gas due to thermal decomposition. On the other hand, under high temperatures, the pore structure of thermal insulation material will first be destroyed, leading to local densification and serious sintering. It will accelerate the leakage of phase change materials and fail the function of thermal insulation.

Significant advances in the structure evolution of silica aerogel has been studied. For example, Lei et al. [29] experimentally studied the structural characteristics at different temperatures and thermal conductivities of silica aerogel. Due to a limited observation method, it is difficult to obtain the structural evolution at the nanoscale. Therefore, the evolution process of silica aerogel at high temperatures was observed by in-site heating TEM. The three stages of the sintering process of aerogel were proposed. Despite the above advances, it is still insignificant to obtain more details about silica aerogel at high temperatures. An atomic-level simulation was proposed by Yang et al. [30]. They analyzed the sintering process of silica aerogels by molecular dynamics simulation. It was found that surface diffusion occurred at the nanoparticle surface; then it turned into volume diffusion to further destroy the backbone structure. However, the structural evolution of silica aerogel with faults is not considered in the reference.

It is known that the neighboring silica backbone will no doubt be affected by the existing faults. Those faults will damage the properties of silica aerogels. Patil et al. found that the faults will significantly reduce the mechanical properties of silica aerogel and its composites [31]. Under high temperatures, the silica backbone with faults may perform a decrease in thermal stability. Those fault effects may be pronounced as the increase in the degree of faults. However, this structural evolution process at high temperature occurs at the nanoscale. It is difficult to observe through experimental measurement, which is also addressed in this work.

In this work, the evolution process for fault silica aerogels under different temperatures is given in Section 2. The number density distribution for the evolution process is given in Section 2. A brief conclusion is shown in Section 3. A molecular dynamics simulation setting, including models of silica aerogel with faults, and atomic interaction settings are proposed in Section 4.

## 2. Results and Discussion

### 2.1. Evolution Process for Fault Silica Aerogel under Different Temperatures

With regard to fireproof materials, it is necessary to evaluate their thermal stability, especially, silica aerogel with faults. In this section, the evolution process of silica aerogel with different faults (10%, 20%, 30%, and 40%) at 500, 800, 1000, and 1500 K is proposed.

**Figure 1:**
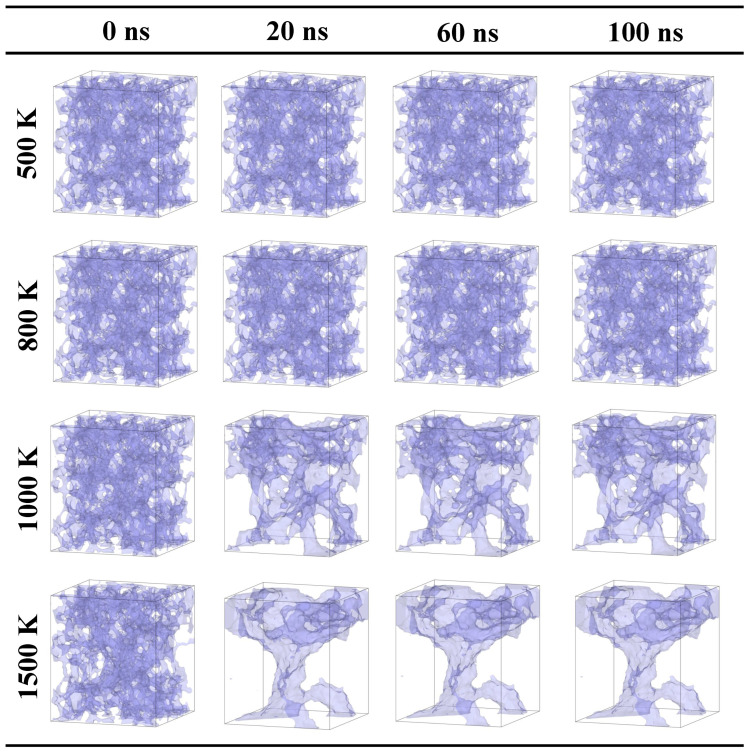
Snapshots of the evolution process at different temperatures for the case with a tensile strain of 10% (23 × 23 × 25.2$\;{\mathrm{nm}}^{3}$).

As shown in Figure 1, for cases with a tensile strain of 10%, the system is coupled with an NVT ensemble for 100 ns at temperatures of 500, 800, 1000, and 1500 K. When the temperature is 500 K, as the heating time increases, it is difficult to observe the fracturing and sintering. The silica backbone is thermally vibrated. As the temperature increases to 800 K, the same trend is found. It indicates that the effects of faults are limited in the structural change in silica aerogel (tensile strain of 10%) when the temperature is less than 800 K.

However, as the temperature is 1000 K, the local densification is observed after 20 ns. The small pore structures merge into a denser silica backbone. As simulation time increases over 60 ns, the further densification of the silica backbone is not observed. When the temperature increases to 1500 K, the silica backbone is much more denser than that in 1000 K. The pore structure around the silica backbone with a different thickness is observed. It can be remarked as the “inhomogeneous solid backbone”. Apparently, the thicker silica backbone performs slighter thermal vibration than that of the thinner backbone. Those different degrees of thermal vibration will destroy the silica backbone, especially the thinner one, forming a denser structure.

In summary, when the tensile strain is 10%, the limited effect of faults on the structural change of silica backbone as temperature increases is observed. The thermal vibration dominates the silica backbone structure.

**Figure 2:**
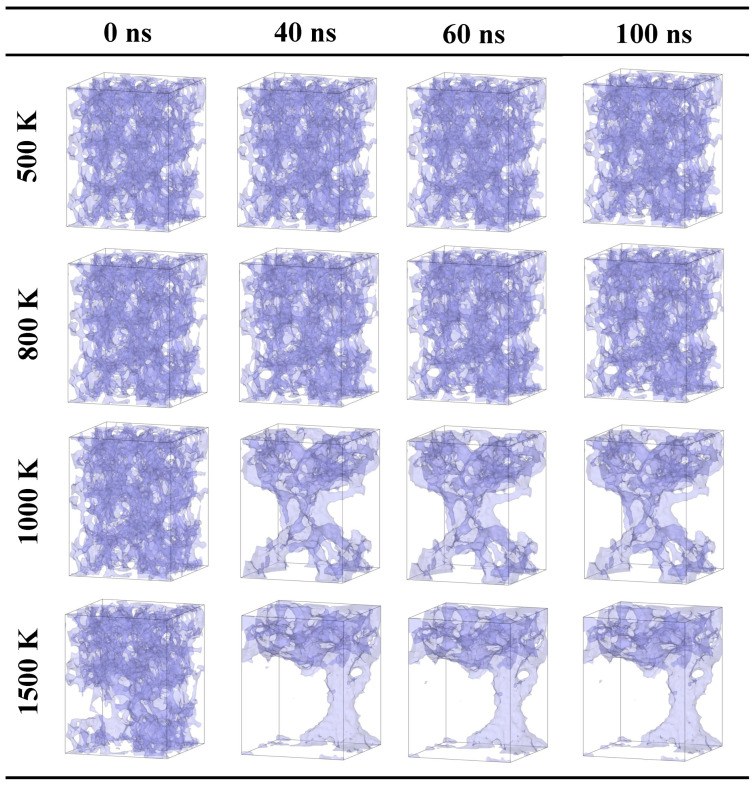
Snapshots of the evolution process at different temperatures for the case with a tensile strain of 20% (21.2 × 21.2 × 25.7$\;{\mathrm{nm}}^{3}$).

As the tensile strain of silica aerogel reaches 20%, the apparent change in the pore structure of the system is observed (as shown in Figure 2). When the temperature is 500 K, the silica structure is kept thermally vibrating. As the temperature increases to 800 K, the pore structure is still kept stable. It indicates that the natural faults show limited effects in the structural change. However, as temperature reaches 1000 K, an obvious fracturing is observed along the natural faults, when the simulation is over 40 ns. Compared with cases with a tensile strain of 10%, the thickness of the silica backbone around the pore structure is more inhomogeneous than that in the case with a tensile strain of 10% due to the natural faults. Under serious sintering, those “inhomogeneous effects” will accelerate the thermal vibration. It can be observed that more fractures occur at 20 ns. As temperature reaches 1500 K, it is clear to see that the silica solid backbone merges along the natural faults.

In summary, when the tensile strain of system is 20%, the local solid backbone thickness is more inhomogeneous. The limited effects are observed when the temperature is less than 800 K. However, as the temperature is over 1000 K, the solid backbone merges and expands along the natural faults. As the temperature reaches 1500 K, the serious vibration will lead to a significant shrinkage.

**Figure 3:**
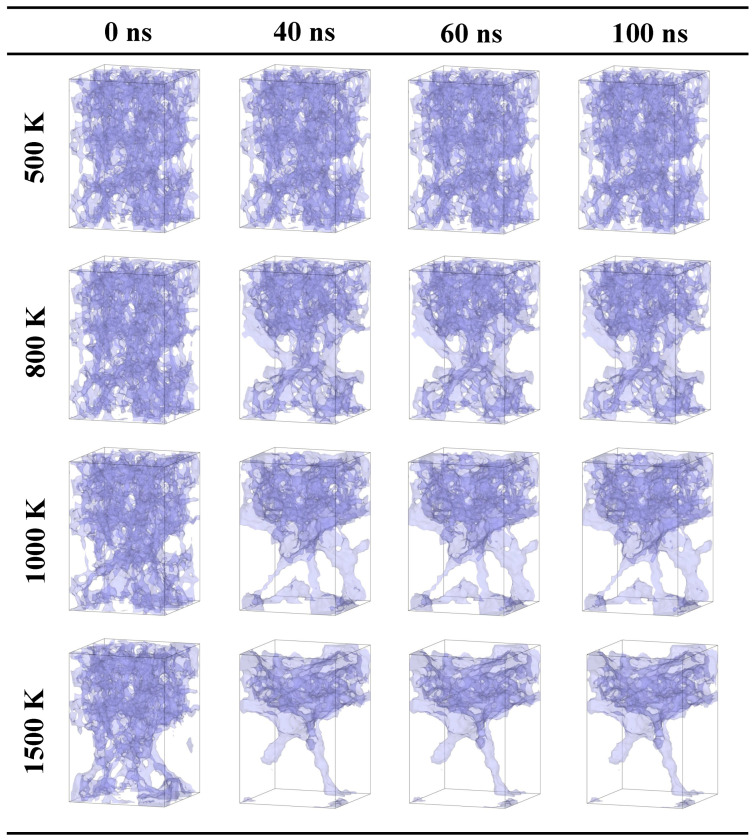
Snapshots of the evolution process at different temperatures for the case with a tensile strain of 30% (20.1 × 20.1 × 29.8$\;{\mathrm{nm}}^{3}$).

When the tensile strain of a system is 30% (as shown in Figure 3), it indicates an obvious fracturing in the silica backbone. It can be observed that the silica backbone is kept stable and vibrates thermally at 500 K. As the temperature increases to 800 K, the serious vibration of the silica backbone results in further fracturing. It can be observed that the silica backbone close to the faults becomes thicker than the other silica backbone. It indicates that the local silica backbone is merging. Compared with the case with a tensile strain of 20%, the local densification of the silica backbone is observed at 800 K. As the temperature reaches 1000 K, the mass transfer of the silica backbone from a thin backbone to a thick backbone is found. A denser silica backbone with a pore structure is observed. The silica backbone close to natural faults sinters seriously. When the temperature is 1500 K, the denser silica backbone further extends and merges with other silica backbones.

In summary, when the tensile strain of a system is 30%, the effects of natural faults cannot be neglected. When the temperature reaches 800 K, an obvious sintering of the silica backbone is found along the faults. When the temperature is over 1000 K, the porous silica backbone is failed.

**Figure 4:**
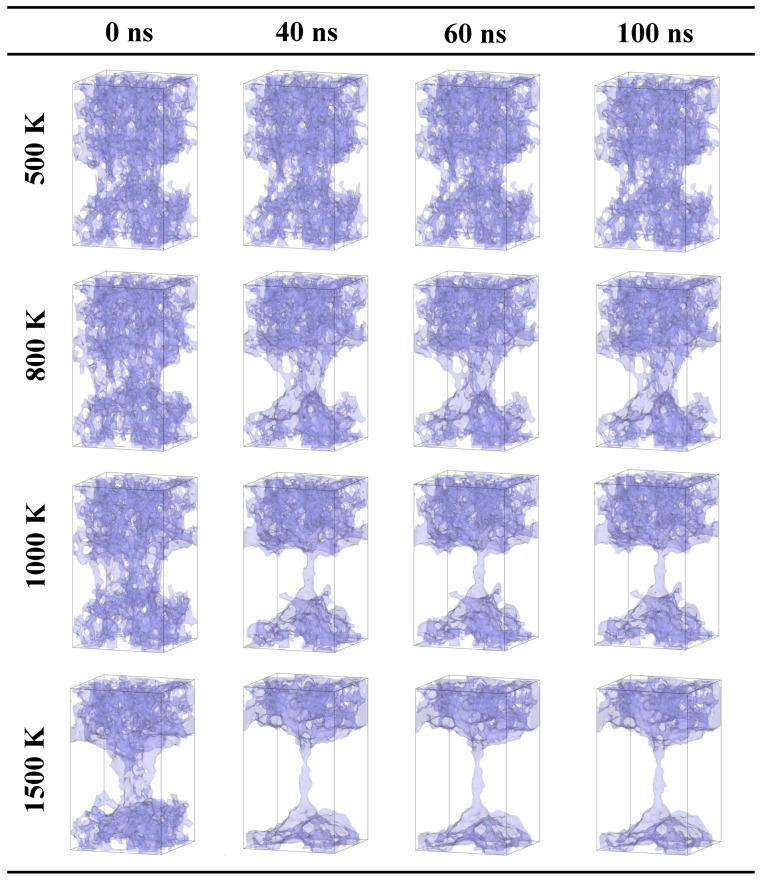
Snapshots of the evolution process at different temperatures for the case with a tensile strain of 40% (18.9 × 18.9 × 32.1$\;{\mathrm{nm}}^{3}$).

As shown in Figure 4, as the tensile strain of a system reaches 40%, it indicates a serious structural fracturing. When the temperature is 500 K, an obvious fracture of a system is observed. The system thermally vibrates at 500 K. Further fracturing and merging is not found. As the temperature is 800 K, the silica backbone close to the faults merges into a denser silica cluster after 20 ns. When the temperature increases to 1000 K, the denser silica cluster extends along the silica backbone and the mass transfer is found. The silica backbone fractures. As the temperature increases to 1500 K, the silica backbone further merges. Additionally, the function of a porous silica backbone as flame-retardant material is failed. In summary, for the case with a tensile strain of 40%, when the temperature is lower than 500 K, the silica backbone can sustain the stability. However, when the temperature is over 800 K, an obvious fracturing induced by faults is observed.

Based on the above analysis, when the temperature is lower than 1000 K and the tensile strain is lower than 20%, the silica backbone thermally vibrates at a high temperature. However, when the tensile strain is over 30%, the effects of faults on the structure change of the silica backbone cannot be neglected.

### 2.2. Effects of Faults on the Thermal Stability of Aerogel

Based on the above analysis, it is still difficult to obtain more details about how the silica backbone merges at a high temperature. Additionally, it is not clear whether the effects of faults are on the thermal stability of silica aerogel. The local densification and evolution process of silica atoms close to the faults cannot be directly obtained by the three-dimensional atomic model for silica aerogel. Therefore, in this section, the number density distribution of the X–Z axis for each case is calculated.

**Figure 5:**
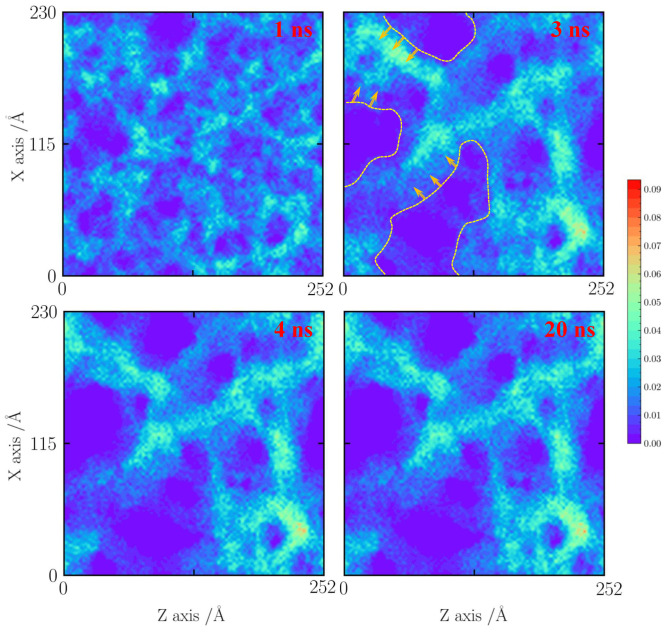
Number density distribution for the case with a tensile strain of 10% at 1000 K.

For the case with a tensile strain of 10%, as shown in Figure 5, the cases at 1000 and 1500 K are shown here. Because the temperature is lower than 1000 K, the effects of faults are neglected. When the time is less than 1 ns, it can be observed that the silica backbone structure is formed by a mixed pore structure including sub-pores (less than 1 nm) and pores (about 3 nm). An obvious shrinkage of a thin silica backbone is found. The 1 nm pore structures are almost destroyed, forming as a big silica cluster. As the simulation time is over 4 ns, the silica backbone becomes a dense structure. After 20 ns, the silica backbone remains stable.

**Figure 6:**
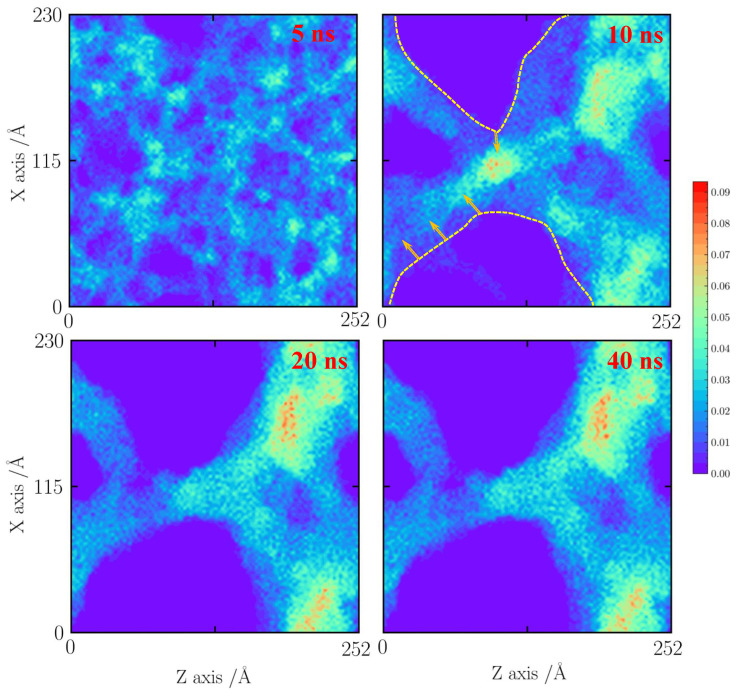
Number density distribution for the case with a tensile strain of 10% at 1500 K.

As the temperature increases to 1500 K, as shown in Figure 6, the pore structures thermally vibrate and further merge into the more dense silica cluster at 10 ns. When the simulation time increases to 20 ns, the mass transfer process from the thin silica backbone to the silica cluster is found. After 20 ns, the structure remains stable.

**Figure 7:**
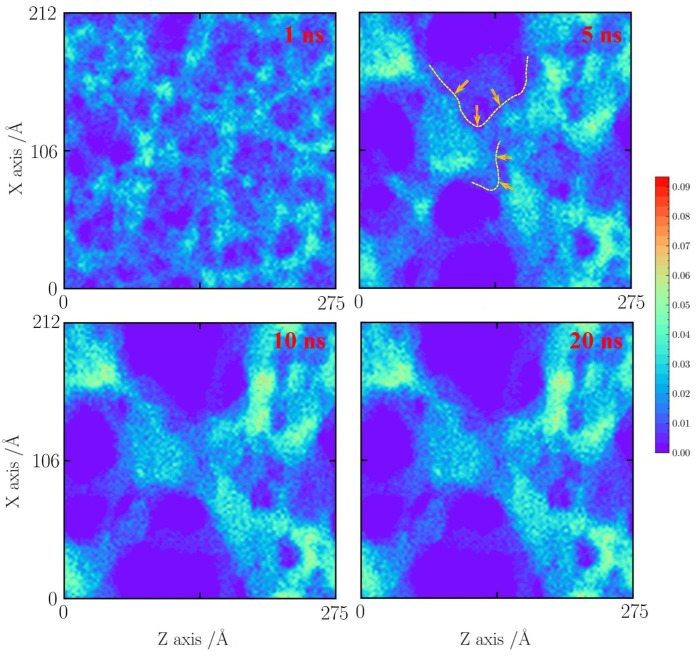
Number density distribution for the case with a tensile strain of 20% at 1000 K.

**Figure 8:**
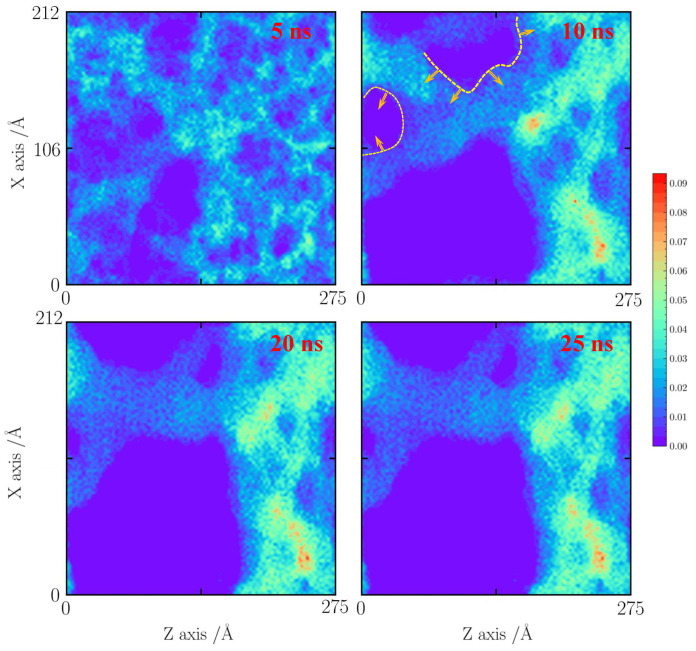
Number density distribution for the case with a tensile strain of 20% at 1500 K.

For the cases with a tensile strain of 20%, the cases at 1000 and 1500 K are calculated, as shown in Figure 7 and Figure 8. The cases at 500 and 800 K show the structure stability.

As shown in Figure 7, with a tensile strain of 20%, it seems that the silica backbone merges along the natural faults and extends to the neighboring silica backbone. At 5 ns, the local densification of the silica backbone is found. The silica cluster is formed. At 10 ns, the silica backbone remains stable.

As the temperature increases to 1500 K, the faults will enhance the merging process of the silica backbone. The pore structure will form a large silica cluster at 10 ns. The small pore further shrinks, leading to the fracturing of the silica backbone.

**Figure 9:**
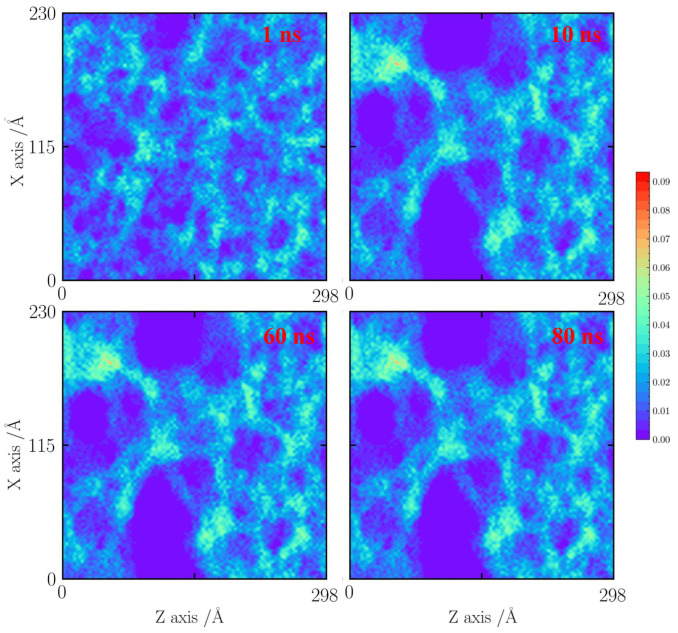
Number density distribution for the case with a tensile strain of 30% at 800 K.

**Figure 10:**
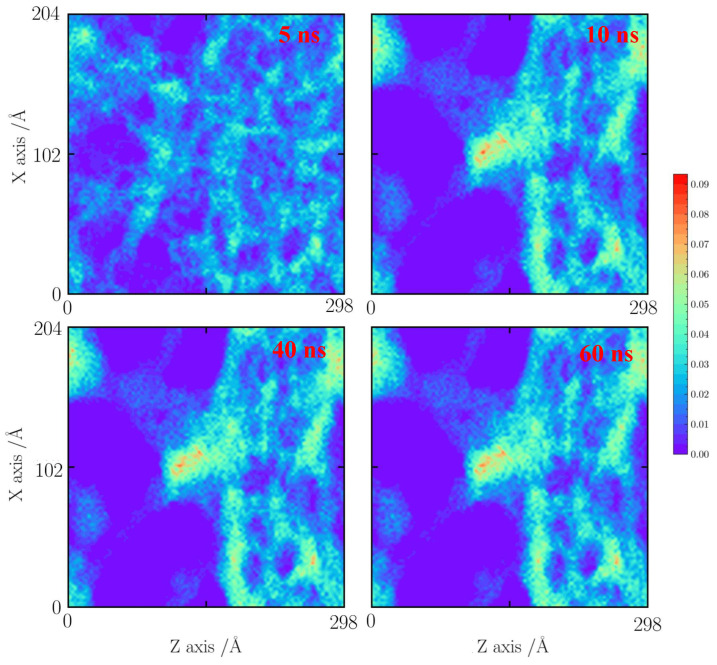
Number density distribution for the case with a tensile strain of 30% at 1000 K.

**Figure 11:**
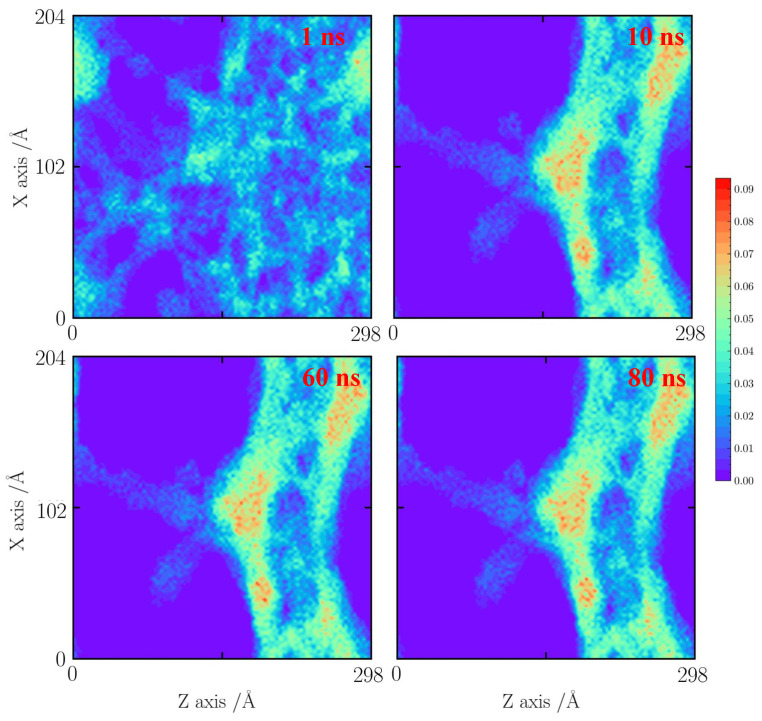
Number density distribution for the case with a tensile strain of 30% at 1500 K.

For the cases with a tensile strain of 30%, the cases at 800, 1000, and 1500 K are used for comparisons. An obvious silica backbone fracture is observed at 1 ns, as shown in Figure 9, Figure 10 and Figure 11. At 800 K, the silica backbone neighboring the faults merges and forms a big cluster. It results in a fault extension at 10 ns. Then, the silica structure remains stable after 60 ns.

When the temperature reaches 1000 K (Figure 10), it can be observed that the silica backbone merges seriously to form a dense silica cluster at 10 ns. Only a small silica backbone remains stable. When the temperature increases to 1500 K (Figure 11), the silica backbone is suddenly destroyed to form a dense silica film at 60 ns.

**Figure 12:**
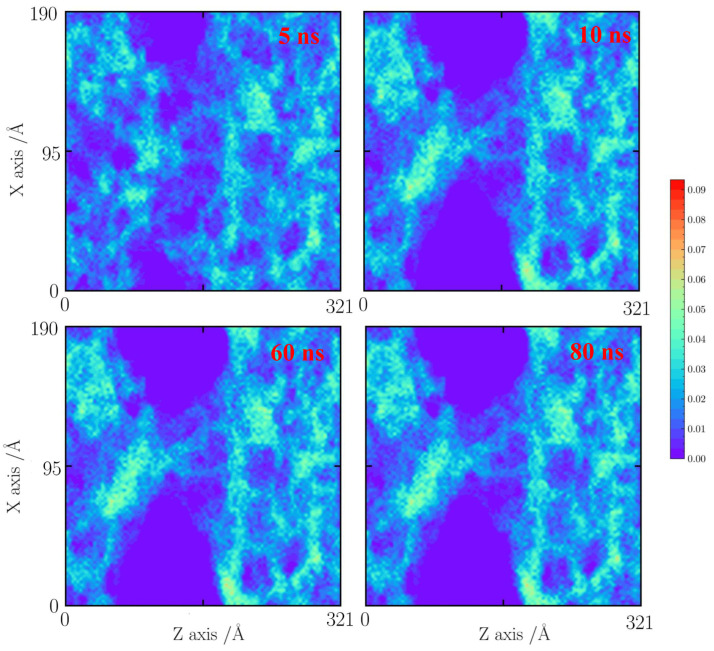
Number density distribution for the case with a tensile strain of 40% at 800 K.

**Figure 13:**
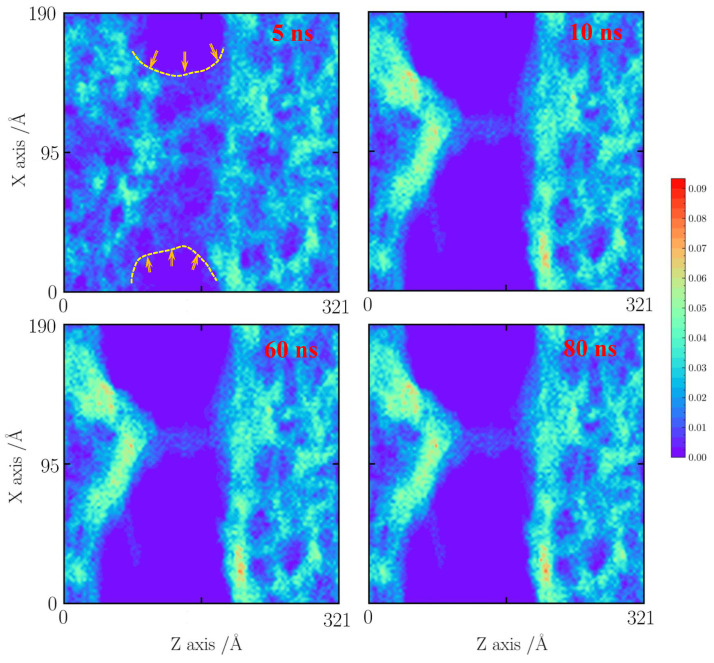
Number density distribution for the case with a tensile strain of 40% at 1000 K.

**Figure 14:**
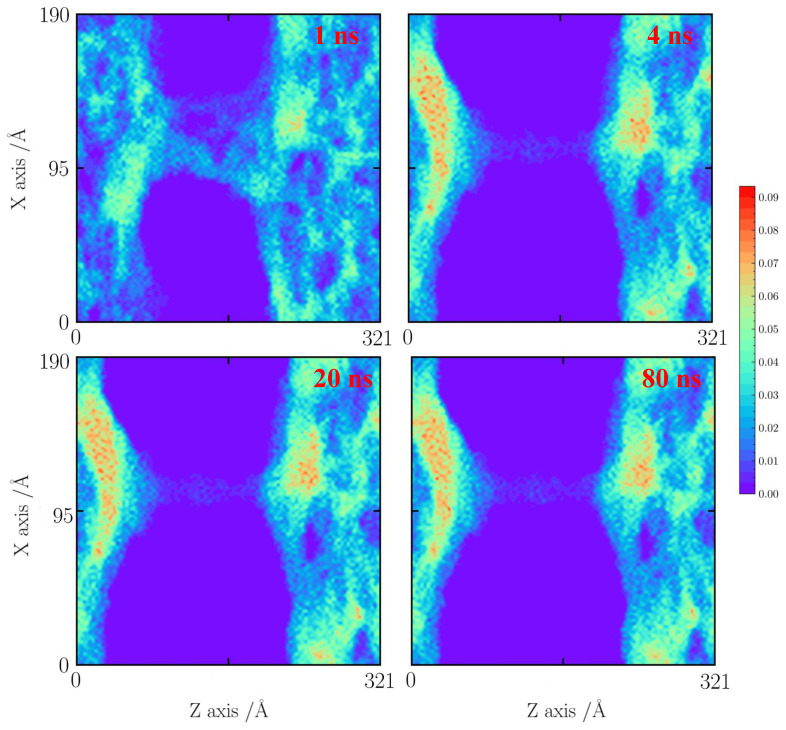
Number density distribution for the case with a tensile strain of 40% at 1500 K.

As shown in Figure 12, Figure 13 and Figure 14, for the cases with a tensile strain of 40%, the number density distributions of cases with 800, 1000, and 1500 K are provided here. As shown in Figure 12, at 800 K, the silica backbone fractures along the faults by thermal vibration. Few silica backbones are connected with each other.

As shown in Figure 13, as the temperature increases to 1000 K, the mass transfer is found. The thin silica backbone moves into the neighboring silica cluster. The silica backbone is sliced into two silica films. As shown in Figure 14, the silica backbone further merges into a dense silica cluster. An increase in local density can be observed at 80 ns.

In summary, the effects of faults for thermal stability can be divided into the following two stages:

(1) When the tensile strain is less than 20%, the silica backbone remains stable at 800 K. The silica merging is caused by the thermal vibration. At over 1000 K, the local densification is enhanced, leading to mass transfer.
(2) When the tensile strain is less than 30%, the silica backbone can only remain stable at 500 K. The faults will cause the structure to fracture. At over 1000 K, a sudden fracture is found, and the silica backbone is failed.

### 2.3. The Role of Faults

Based on the above analysis, two questions are raised: (1) What is the role of heat treatment as temperature increases? (2) How can it be understood that, with less strain (<20%), silica aerogel remains stable at 800 K but fails at a strain of 30%?

**Figure 15:**
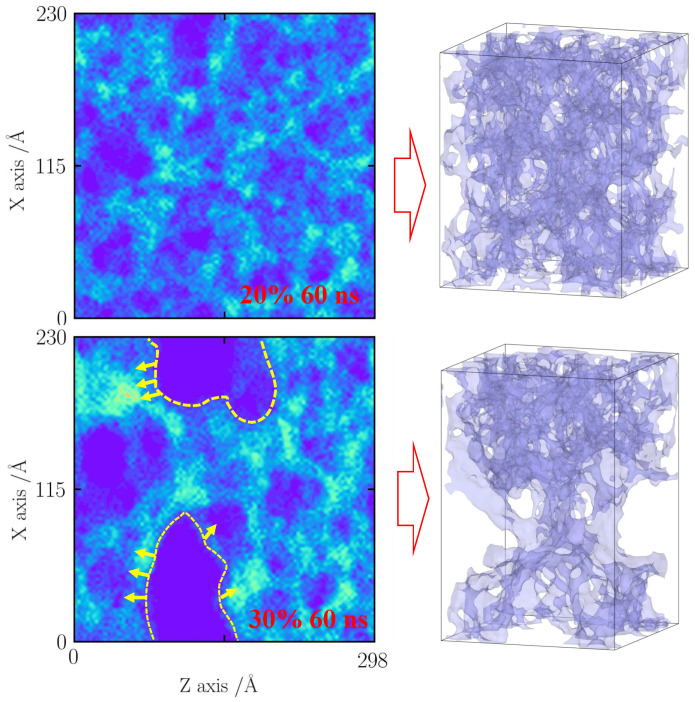
Effects of faults: silica aerogel with a strain of 20% vs. a strain of 30% at 800 K and 60 ns.

To answer the above-mentioned questions, as shown in Figure 15, with a strain of 20%, the small pore of silica aerogel is kept stable. In other words, silica aerogel thermally vibrates at 800 K. However, as the strain increases to 30%, it indicates a large fracture. The silica close to these faults will enhance the local thermal vibration. It results in the silica close to the faults more easily merging as a local densification. Then, the local silica densification will decrease its thermal vibration. The silica backbone close to the faults will be easily destroyed due to the local inhomogeneous vibration until a thick silica backbone forms. Based on the density distribution, an increase in local density close to the faults is observed. Therefore, heat treatment results in the silica backbone vibration damaging the thin silica backbone due to inhomogeneous vibration. Additionally, for the case with a strain of 30%, the faults will induce further merging for the silica backbone structure close to the faults.

## 3. Conclusions

In this work, the structure evolution of silica aerogel with faults at a very high temperature is studied through large-size molecular dynamics simulation. An atomic model of silica aerogel with 0.48 g/cm^3^ is built. Additionally, the faults in silica aerogel are generated by the tensile process to generate the random sub-pores. The structure evolution process of silica aerogel with faults at different temperatures (500, 800, 1000, and 1500 K) is studied. It is found that, at a tensile strain of 10%, the effects of faults on the thermal stability of silica aerogel are limited. The thermal vibration will dominate the heating process. At 1500 K, the function of silica aerogel is failed. At a tensile strain of 20%, the silica aerogel structure remains stable, indicating the dominant role of thermal vibration during the heating process, when the temperature is less than 800 K. The silica backbone merges and fails at 1000 K. When the tensile strain of a system is over 30%, the faults will enhance the merging process. An obvious densification in the silica backbone is found. The silica backbone can only remain stable at 800 K.

Methodologically, to simulate the heating process and obtain the structure evolution process, a large simulation time of 100 ns is used. It should be noted that the silica aerogel model is over 23 nm. It is believed that these results will inspire more design for fire-retardant and safety building materials.

## 4. Materials and Methods

**Figure 16:**
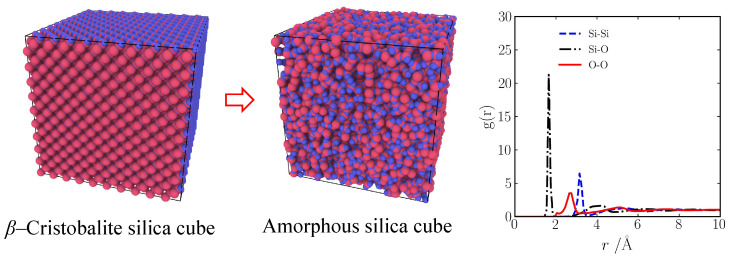
Amorphous silica model and its radial distribution function.

In this work, all molecular dynamics simulations are used by an open-source Large-Scale Atomic/Molecular Massively Parallel Simulator (LAMMPS) [32]. First, the atomic model of silica aerogel is built using the method of “negative rupturing”. The amorphous silica is first built starting with the β-cristobalite (density is 2.17 g/cm^3^) by the annealing process. The system is periodic boundary conditions, and the time step is 0.5 fs. The velocity Verlet algorithm in used to assign the velocity for atoms at 7000 K. Subsequently, the sample was quenched to 300 K using an NVT ensemble to obtain amorphous silica. It is validated by a radial distribution function, as shown in Figure 16. The details can be found in ref. [11,33]. Then, the amorphous silica sample was relaxed at 300 K for 7.5 ps. The system was expanded to the desired density and heated to 3000 K for 50 ps. Finally, the system was quenched to 0 K by energy minimization. The details can be found in ref. [34].

The faults in silica aerogel are generated by the strain tensile. The model of silica aerogel is coupled with an NVT ensemble for 1 ns. Then, the strain rate is set to 0.0004 $ps^{-1}$ until the system along the Z-axis reaches the strains of 10%, 20%, 30%, and 40%, respectively.

It should be noted that silica–silica interactions are described by Tersoff potential composed of the two-body terms [35], expressed as

(1) $$\begin{array}{c} E=\sum_{i}E_{i}=\frac{1}{2}\sum_{i\neq j}V_{ij} \end{array}$$

(2) $$\begin{array}{c} V_{ij}=f_{c}\left(r_{ij}\right)\left[f_{R}\left(r_{ij}\right)+b_{ij}f_{A}\left(r_{ij}\right)\right] \end{array}$$

(3) $$\begin{array}{c} f_{R}\left(r_{ij}\right)=A_{ij}\exp\left(-\lambda_{ij}r_{ij}\right) \end{array}$$

(4) $$\begin{array}{c} f_{A}\left(r_{ij}\right)=B_{ij}\exp\left(-\mu_{ij}r_{ij}\right) \end{array}$$

(5) $$f_{c}\left(r_{ij}\right)=\left\{\begin{array}{cc} 1, & r_{ij}<R_{ij}\\ \frac{1}{2}+\frac{1}{2}\cos\left(\pi \frac{r_{ij}-R_{ij}}{S_{ij}-R_{ij}}\right), & R_{ij}<r_{ij}<S_{ij}\\ 0, & r_{ij}>S_{ij} \end{array}\right.$$

(6) $$\begin{array}{c} b_{ij}=\chi_{ij}{\left(1+\beta_{i}^{n_{i}}\zeta_{ij}^{n_{i}}\right)}^{-1/2n_{i}} \end{array}$$

(7) $$\begin{array}{c} \zeta_{ij}=\sum_{k\neq i,j}f_{c}\left(r_{ik}\right)\omega_{ik}g\left(\theta_{ijk}\right) \end{array}$$

(8) $$\begin{array}{c} g\left(\theta_{ijk}\right)=1+c_{i}^{2}/d_{i}^{2}-c_{i}^{2}/\left[d_{i}^{2}+{\left(h_{i}-\cos\theta_{ijk}\right)}^{2}\right] \end{array}$$

(9) $$\begin{array}{c} \lambda_{ij}=\left(\lambda_{i}+\lambda_{j}\right)/2,\;\mu_{ij}=\left(\mu_{i}+\mu_{j}\right)/2 \end{array}$$

(10) $$\begin{array}{c} A_{ij}={\left(A_{i}+A_{j}\right)}^{1/2},\;B_{ij}={\left(B_{i}+B_{j}\right)}^{1/2} \end{array}$$

(11) $$\begin{array}{c} R_{ij}={\left(R_{i}R_{j}\right)}^{1/2},\;S_{ij}={\left(S_{i}S_{j}\right)}^{1/2} \end{array}$$

where *i*, *j*, and *k* are the atomic labels; $r_{ij}$ is the bond length between the atoms *i* and *j*; and $\theta_{ijk}$ is the bond angle between the ij and ik bonds. The parameter $\chi_{ij}$ in Equation (6) is used for the weakening or strengthening of heteropolar bonds. The charge transfer between Si and O is also taken into account through the parameter $\chi_{Si-O}$. $\omega_{ik}$ in Equation (7) can permit greater flexibility. The parameters $\lambda_{ij}$, $\mu_{ij}$, $A_{ij}$, $B_{ij}$, $R_{ij}$, and $S_{ij}$ are the subscripted parameters depending on the atomic types. The details can be found in ref. [35].

An atomic model (23 × 23 × 23$\;{\mathrm{nm}}^{3}$, 192,000 atoms) of a porous silica backbone with a density of 0.48 g/cm^3^ is obtained. Then, the system is strained to 10% (23 × 23 × 25.2$\;{\mathrm{nm}}^{3}$), 20% (21.2 × 21.2 × 25.7$\;{\mathrm{nm}}^{3}$), 30% (20.1 × 20.1 × 29.8$\;{\mathrm{nm}}^{3}$), and 40% (18.9 × 18.9 × 32.1$\;{\mathrm{nm}}^{3}$) along the Z-axis to generating the random faults during a tensile process. Then, to quantitatively measure the effects of faults on the thermal stability of silica aerogel, a large simulation time is set as 100 ns with time steps of 0.01 ps. The system is coupled with an NVT ensemble for 100 ns at different temperatures of 500, 800, 1000, and 1500 K.

## Data Availability

The data presented in this study are openly available in article.
